# A Fixed‐Charge Interphase Synchronizes Ion Transport to Suppress Space‐Charge‐Driven Inefficiency Under Nanoliter Confinement

**DOI:** 10.1002/anie.5195226

**Published:** 2026-06-30

**Authors:** Yaping Yan, Jiachen Ma, Wenlan Zhang, Hongmei Tang, Yue Li, Ruhuai Mei, Yang Huang, Daniil Karnaushenko, Dmitriy D. Karnaushenko, Yumin Luo, Kai Zhang, Oliver G. Schmidt, Minshen Zhu

**Affiliations:** ^1^ Research Center for Materials Architectures, and Integration of Nanomembranes (MAIN) Chemnitz University of Technology Chemnitz Germany; ^2^ Material Systems for Nanoelectronics Chemnitz University of Technology Chemnitz Germany; ^3^ Sustainable Materials and Chemistry Department of Wood Technology and Wood‐based Composites University of Göttingen Göttingen Germany; ^4^ Advanced Materials Thrust Function Hub The Hong Kong University of Science and Technology (Guangzhou) Guangzhou Guangdong China

**Keywords:** battery system, chemical physics, Debye length, electrolyte, Faraday efficiency, ion, materials science, microfabrication, microscale chemistry

## Abstract

Ion transport at electrified interfaces is conventionally described by the redistribution of mobile ions to preserve local electroneutrality. Under extreme electrolyte confinement, however, this assumption fails as the characteristic transport length approaches the Debye screening length, giving rise to space‐charge accumulation and slow electrostatic relaxation that dominate interfacial kinetics. Here, we introduce a fixed‐charge‐selective interphase in which immobile anionic charges replace mobile electrolyte anions as the primary charge‐compensating species, thereby establishing a chemically encoded electrostatic boundary condition. Using a glucose‐derived network as a model system, we show that localized fixed charge enables cation‐selective transport and suppresses extended space‐charge layers (ESCLs) by eliminating the slow relaxation pathways. Spatiotemporal transport analysis reveals that this interphase collapses multi‐timescale interfacial relaxation into a unified kinetic regime. When applied to nanoliter‐confined electrochemical systems (45 nL), rest‐induced Coulombic efficiency (CE) collapse is reduced from 40% to 5%, demonstrating stabilization of electrostatic relaxation during idle periods, which is a failure mode intrinsic to microscale devices operating under duty cycles. The concept is further validated under pH‐coupled and oxidative‐stress conditions, sustaining stable operation with strong rate capability. These results define a general chemical strategy for regulating interfacial ion transport under confinement by replacing mobile charge compensation with molecularly fixed charges.

## Graphical abstract

Electrochemical energy storage breaks down under extreme miniaturization, where interfaces and space‐charge dominate device behavior. This work introduces a fixed‐charge selective interphase (FCSI) integrated into a twin‐tube micro‐Swiss‐roll architecture. By coupling interphase chemistry with device geometry, the resulting highly reversible Zn anode allows the full microcells to operate stably with nanoliter‐scale electrolytes, establishing a new strategy for reliable microscale energy storage.

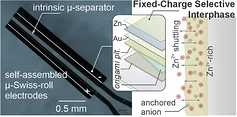

## 1 Introduction

**FIGURE 1:**
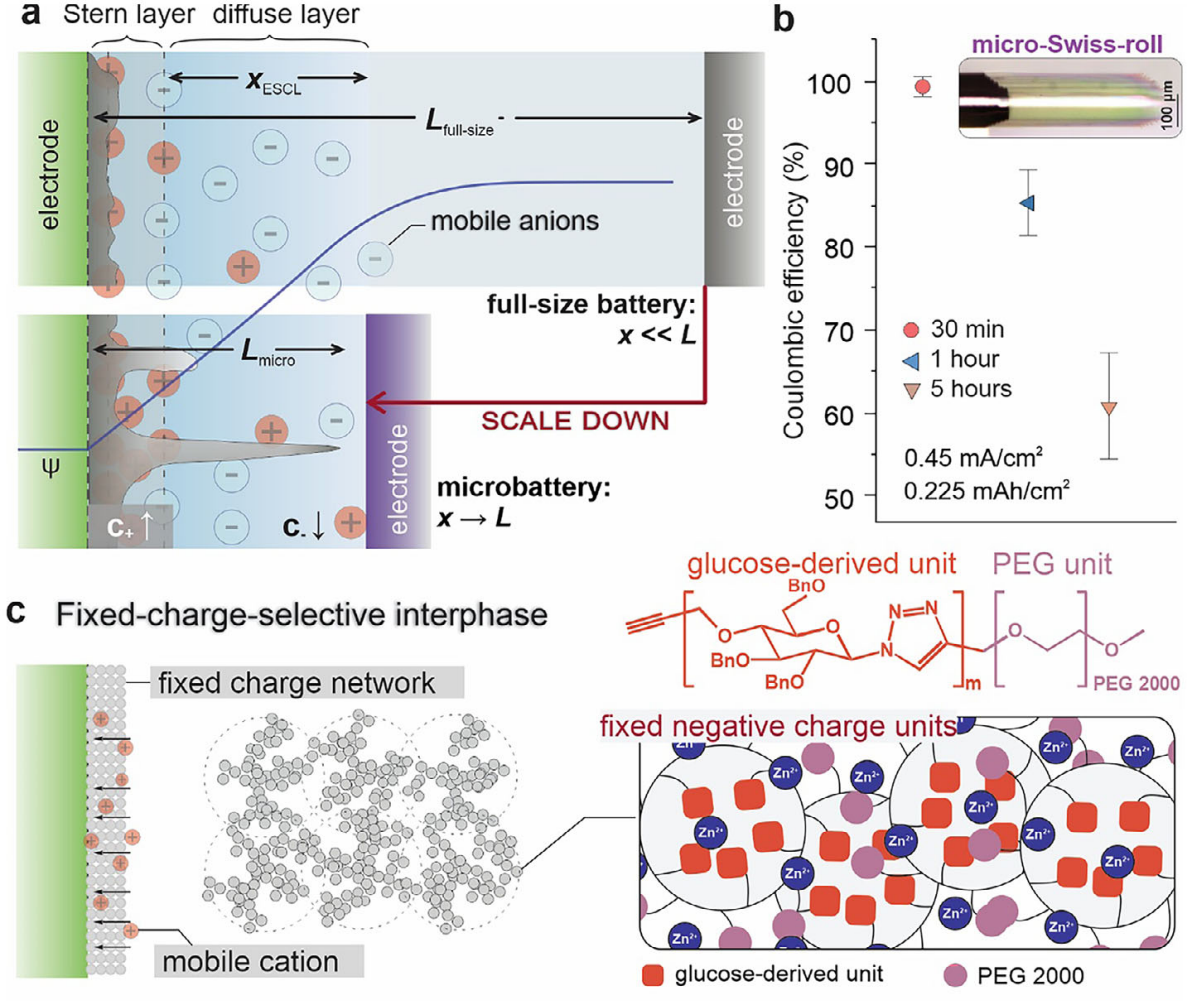
Space‐charge dominance under microscale confinement and chemical substitution of charge compensation. (a) Scaling analysis showing ESCL thickness (*x*) approaching interelectrode distance (*L*) under microscale confinement, breaking local electroneutrality and creating device‐spanning electric fields. (b) Rest‐induced Coulombic efficiency loss. The inset shows a micro‐Swiss‐roll electrode (scale bar 100 µm). (c) Schematic illustration of FCSI with fixed “anions” for charge compensation.

Ion transport at electrified interfaces underpins processes for electrochemical energy storage. Classical descriptions assume that charge compensation is achieved through the redistribution of mobile ions within the electrolyte, giving rise to electric double layers that preserve local electroneutrality (Figure 1a, upper panel). As characteristic dimensions shrink toward the sub‐millimeter regime, interfaces no longer represent a localized perturbation but instead dominate the entire electrochemical system [1]. In particular, ion depletion near electrodes leads to the formation of extended space‐charge layers (ESCLs) at the electrode–electrolyte interface [2, 3]. Strategies for harnessing or suppressing ESCLs have been explored across several domains of electrochemistry [4, 5, 6, 7]. In bipolar membranes (BPMs), the junction between cation‐ and anion‐exchange layers creates an intrinsic space‐charge region [8, 9, 10]. This depleted junction governs the membrane's ability to dissociate water under reverse bias, which is central to electrodialysis, CO_2_ electrolysis, and artificial photosynthesis [11, 12]. Nanometer‐thick, layer‐by‐layer‐assembled interlayers achieve similar effects without disrupting the second Wien effect contribution, because the catalyst thickness remains comparable to the space‐charge width [13, 14]. Recent work has further shown that ion solvation kinetics at the BPM junction and at electrolyte|metal interfaces share a common physics: both are governed by bias‐dependent interfacial capacitance and the excess charge needed to restructure interfacial water [11, 15]. An analogous situation arises in nanofluidic devices, where confined channels lined with fixed surface charges or polyelectrolyte layers exhibit overlapping electrical double layers that eliminate the bulk solution phase entirely. Under these conditions, ion transport becomes surface‐charge‐governed rather than bulk‐diffusion‐limited, enabling phenomena such as ionic current rectification, ion selectivity, and field‐effect transistor‐like gating of ionic currents. In such confined channels, polyelectrolyte brush layers placed within the double‐layer region provide a molecularly defined fixed‐charge environment that modulates the local electrostatic potential, enabling tunable selectivity and transport control [16, 17, 18, 19]. These systems demonstrate a general principle: inserting fixed or quasi‐fixed charges into a space‐charge‐dominated region can replace the dynamic mobile‐ion redistribution that normally governs electroneutrality, fundamentally changing transport behavior [20].

Electrochemical energy storage is a domain in which the physics of space‐charge confinement is becoming increasingly critical. Emerging applications in dust‐sized sensors, microrobots, and implantable devices demand batteries with sub‐millimeter footprints and nanoliter‐scale electrolyte volumes [21, 22, 23, 24, 25, 26, 27, 28, 29, 30]. However, unlike microelectronics, where miniaturization often enhances performance (e.g., shortening electron transport pathways) [31], electrochemical energy‐storage systems enter a fundamentally different regime under extreme confinement, which causes classical descriptions to become increasingly fragile. Central to this challenge is metal electrodeposition, which underpins the operation of metal anode batteries (e.g., Zn). Metal anodes are intrinsically susceptible to nonuniform electrodeposition, leading to morphological instabilities such as dendritic growth [32, 33, 34]. This challenge is qualitatively distinct from ion intercalation cathodes, where the host lattice provides structural sites for guest ions and the electrode geometry is largely preserved during cycling. In metal anode systems, however, the electrode surface is continuously created and destroyed, making the electrode|electrolyte interface dynamically evolving and inherently unstable.

The micro‐Swiss‐roll architecture (Figure 1b, inset image) provides a platform for studying these effects under controlled extreme confinement [4, 5, 6]. In this design, thin‐film electrode stacks are deposited on a substrate using microfabrication protocols and then self‐assembled into a tightly wound tubular structure through strain‐driven roll‐up, producing a three‐dimensional electrode within a sub‐millimeter footprint. The resulting interelectrode spacing is on the order of a few micrometers, and the total electrolyte volume is reduced to the nanoliter range (∼45 nL). The thickness of the ESCL can be expressed by the equation: $x\approx L\;{\left\{\left(\frac{Ve}{kT}\right)\left(\frac{\kappa^{-1}}{L}\right)\right\}}^{2/3}$, where *L* is the interelectrode space, *V*/(*kT/e*) denotes electric potential relative to the thermal voltage, *κ* ^−1^ is the Debye screening length, which is inversely proportional to the square root of the ion concentration. The deliberate reduction of *L* renders space‐charge regions extends across the entire device (Figure 1a, bottom panel), corresponding to a breakdown of local electroneutrality, such that net charge density (ρ  =  2*F*(*c*_+_ − *c*_−_) > 0) persists over the entire electrode spacing (− ε∇^2^φ  =  ρ). As a result, the electric field is no longer confined to the interface but spans the device, fundamentally destabilizing ion transport and metal deposition.

Importantly, the consequences of ESCL formation are not limited to nonuniform deposition during active cycling. When external current is interrupted, as occurs during rest or standby operation, the electrochemical system is no longer driven by an applied field but instead evolves under its intrinsic electrostatic and chemical potentials. Under such conditions, ESCLs do not instantaneously collapse. Instead, residual charge accumulation relaxes over extended timescales through mixed‐ion redistribution and parasitic interfacial reactions. This behavior is directly shown in the pronounced rest‐induced Coulombic efficiency (CE) decay observed in Zn metal anodes under confinement to less than 10 µm (Figures 1b and S1). Unlike continuous cycling, where external current maintains a driven steady state, rest periods expose the intrinsic stability of the electrostatic environment by allowing charge redistribution to proceed without external forcing. In conventional interfaces, residual space‐charge regions relax slowly through mixed‐ion migration and parasitic reactions, leading to irreversible charge loss. Nonuniform metal growth accelerates corrosion and hydrogen evolution, promoting the formation of passivating by‐products and electrically isolated “dead points” [35, 36, 37, 38]. The resulting increase in effective surface area further intensifies side reactions, amplifying interfacial heterogeneity and establishing a self‐reinforcing degradation loop: space‐charge‐driven current focusing promotes dendritic growth, while the resulting high‐surface‐area structures accelerate parasitic reactions that further destabilize ion transport and metal deposition As such, CE declines markedly with increasing rest time, falling below 90% after only 1 h and retaining only ∼60% after 5 h.

These considerations reveal a fundamental challenge for highly reversible energy storage under confinement: how to suppress ESCLs. Notably, this challenge cannot be resolved by electrolyte formulation alone, as tuning the bulk ionic composition does not alter the interfacial charge‐compensation mechanism governing space‐charge formation under confinement [39, 40, 41]. Here, we introduce a fixed‐charge selective interphase (FCSI) that addresses this challenge at its electrostatic origin. The FCSI is based on a block copolymer that combines a glucose‐based segment with a polyethylene glycol (PEG) chain, connected through triazole linkages formed by click chemistry (Scheme S1). The glucose units provide densely packed oxygen groups that reversibly coordinate; the triazole nitrogen atoms act as additional anchoring sites that locally enrich Zn^2+^, and the PEG segments offer a flexible, ion‐conducting pathway for Zn^2+^ hopping between coordination sites. These three motifs encode immobile negative charges into the polymer backbone while maintaining fast cation transport, functioning as a synthetic “immobile anion” (Figure 1c) [42, 43, 44, 45]. This design allows charge compensation to be achieved locally and statically, eliminating the need for long‐range anion redistribution that drives space‐charge expansion. Implemented within a micro‐Swiss‐roll electrode (0.44 mm^2^ footprint, inset in Figure 1b) operating under nanoliter‐scale electrolyte conditions (∼45 nL), a glucose‐based molecular network establishes a chemically defined Zn^2+^ coordination environment bridging metal and electrolyte. The resulting FCSI dynamically evolves into a kinetically synchronized solid‐ionic layer, maintaining uniform ion flux and stabilizing Zn deposition. Spatiotemporal transport analysis reveals that fragmented interfacial processes merge into a unified kinetic regime. As a result, the rest‐induced CE loss is reduced from 40% to 5%. The concept of FCSI is further validated by stable operation beyond 250 cycles with strong rate capability in full microbatteries (1.05 mm^2^) paired with MnO_2_ and Prussian‐blue (PB) cathodes in the micro‐Swiss‐roll structure, representing proton‐coupled conversion and high‐voltage oxidative stress conditions, respectively [46, 47, 48]. These results establish fixed‐charge interphases as a design principle for confined electrochemical systems, showing that stabilizing microbatteries requires engineering interfacial electrostatic boundary conditions rather than optimizing bulk transport alone.

## 2 Results and Discussions

The central design principle of replacing mobile ions with fixed charges is to enable selective cation transport without relying on anion redistribution (Figure 1c). This concept naturally parallels biological ion channels, where high selectivity and large ionic flux arise from continuous coordination pathways with low activation barriers. In such systems, ion transport is governed by dynamically polarizable coordination environments that match the ion's preferred coordination energy while maintaining an electronically soft landscape that supports concerted motion. Translating this principle into synthetic materials therefore requires frameworks that combine structural confinement with reversible, multidentate coordination. Carbohydrate‐rich biological matrices provide a compelling blueprint, as dense polyol motifs regulate divalent cations through transient, cooperative binding. Therefore, we opt to use glucose‐derived coordination motifs, forming a dynamic coordination network in which metal–ligand interactions continuously reshape the local electronic environment.

Glucose‐derived building blocks (see nuclear magnetic resonance spectroscopy, Figure S2, and Fourier transform infrared spectroscopy, Figure S3) provide a high density of oxygen donors that yield multidentate, reversible coordination sites for Zn^2+^. N‐donor units in the linker act as anchoring sites that locally enrich Zn^2+^ without inducing irreversible chelation, preventing cation depletion into the bulk electrolyte [49, 50]. Copper(I)‐catalyzed azide–alkyne cycloaddition click chemistry is used to link a glucose‐derived repeat unit to a polyethylene glycol (PEG‐2000) segment (Scheme S1). Ether oxygens along the PEG segments create continuous, low‐barrier pathways for Zn^2+^ hopping, supporting rapid ion redistribution within the interphase [51]. Such an interphase is engineered to replace the role of mobile anions in charge compensation by encoding negative charges (O‐ and N‐donors) into an immobile backbone (an “immobile anion”). Small‐angle x‐ray scattering (SAXS, Figure S4) reveals a surface‐like, fractal network [52, 53]. A compact radius of gyration (*R*_g_ ≈ 9.5 Å) suggests a glucose‐derived core surrounded by PEG chains. The FCSI coating does not increase interfacial resistance, thereby maintaining high electrochemical activity (Figure S5). In contrast, a neutral polymer coating (e.g., PVA) leads to a pronounced decrease in ionic conductivity and is ineffective at suppressing side reactions, as evidenced by the Tafel analysis (Figure S6).

Density functional theory (DFT) calculations confirm the electrochemical stability and coordination characteristics of the interphase (Figure S7). In the absence of Zn^2+^, the isolated glucose‐based monomer exhibits a wide HOMO–LUMO gap of 5.83 eV, ensuring that the interphase does not act as an electronic conductor or redox mediator. The Zn^2^^+^/Zn redox potential (−0.76 V vs. SHE) lies entirely within this gap, indicating that the polymer is electrochemically inert prior to coordination [54]. Moreover, the HOMO is predominantly localized on oxygen‐ and nitrogen‐rich functional groups, indicating the presence of negatively polarized donor sites capable of binding divalent cations. Upon Zn^2+^ coordination, however, the electronic structure undergoes a pronounced reconfiguration, converting the localized lone‐pair orbitals into stabilized bonding states and inducing a pronounced downward shift of the HOMO energy level. This electronic stabilization signifies the formation of strongly bound, negatively charged coordination sites embedded within the polymer backbone. As a result, the local electrostatic environment becomes dominated by spatially fixed charges that selectively attract Zn^2+^ while suppressing anion migration, rendering a Zn^2+^ transference number of 0.62 (Figure S8). Coordination of Zn^2+^ introduces strong orbital hybridization that collapses the HOMO–LUMO gap to 0.28 eV. This indicates a transition from a localized insulating molecular state to a highly polarizable coordination network. Rather than implying increased electronic conduction, this gap collapse reflects strong metal–ligand orbital hybridization that enhances electronic polarizability (Figures S9 and S10). These calculations provide molecular‐level support that the coordination sites are thermodynamically stable within the operating voltage window, reversible rather than irreversibly chelating, and sufficiently polarizable to buffer local charge fluctuations [55].

**FIGURE 2:**
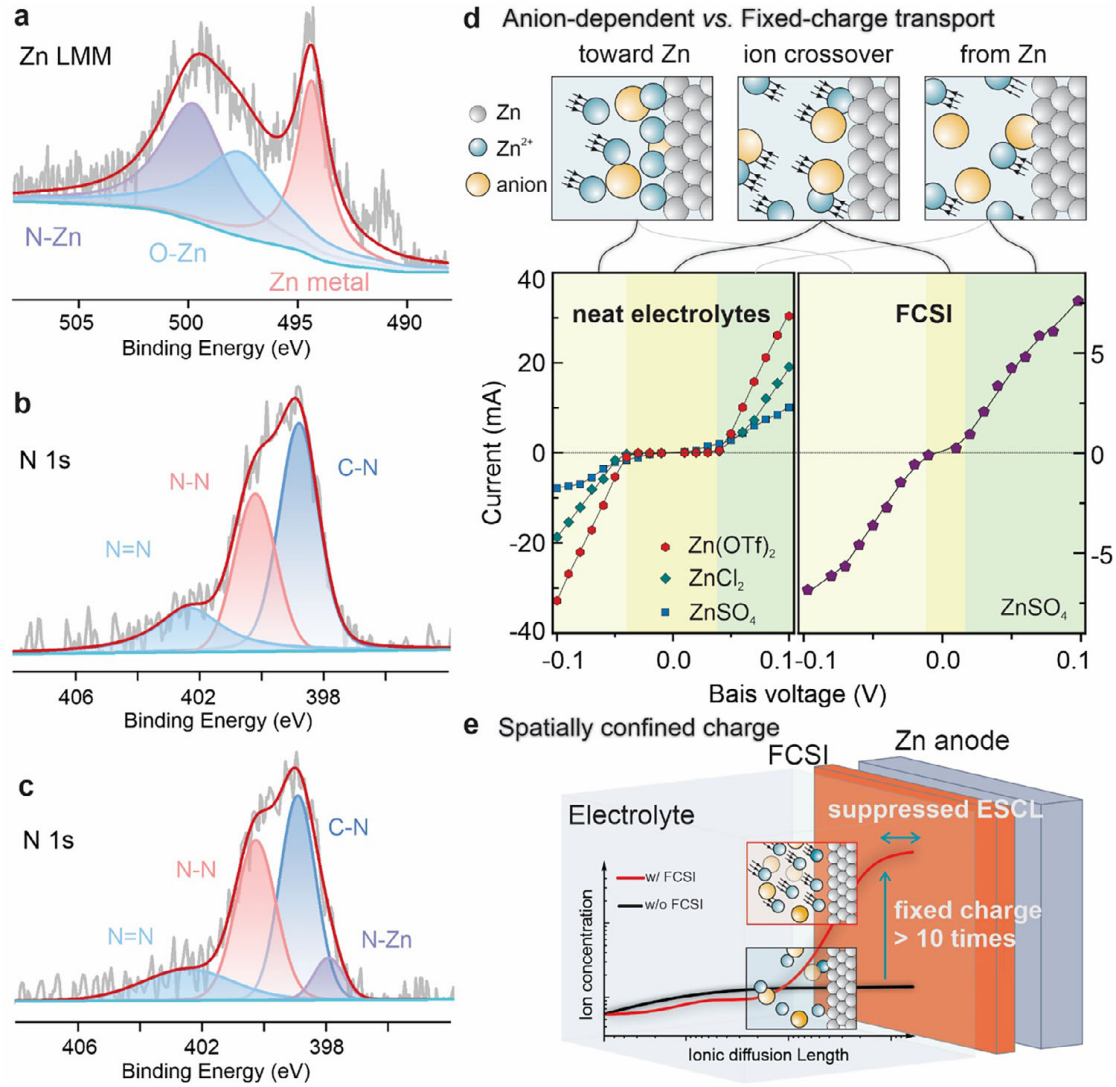
Charge compensation substitution by fixed charges. (a–c) XPS analysis after stripping 25 mAh/cm^2^ Zn: (a) Zn LMM Auger spectra of the FCSI after etching to a depth of 600 nm, and N 1s core‐level spectra for (b) the FCSI|electrolyte interface and (c) FCSI after etching through 600 nm. (d) Schematic and corresponding current–voltage profiles of symmetric cells under bias in (left) neat electrolytes and (right) with FCSI. (e) Ion concentration profiles across (red) electrolyte|FCSI|Zn and (black) electrolyte|Zn stacks.

The electronic‐structure analysis above suggests that the glucose‐derived network is electronically inert in its pristine state, yet becomes selectively responsive upon Zn^2+^coordination through oxygen and nitrogen donor sites. This coordination behavior is consistent with the ion‐exchange function of the interphase. Zn^2+^ ions are not merely electrostatically partitioned into the layer but are locally accommodated through reversible bonding with the polymer backbone. To directly probe this buried interface, we performed depth‐resolved x‐ray photoelectron spectroscopy (XPS) after deeply stripping 25 mAh/cm^2^ of Zn. At the polymer|electrolyte interface, the Zn LMM Auger spectrum showed signals within the spectral noise (Figure S11), indicating a Zn‐free outer surface. After etching through 600 nm of the FCSI, three distinct Zn LMM components emerged at 494.3, 497.6, and 499.7 eV, which are attributed to metallic Zn, Zn–O, and Zn–N species, respectively (Figure 2a) [56, 57]. High‐resolution N 1s spectra supported this assignment: the surface exhibited a triazole peak at 402.3, 400.2, and 398.9 eV (Figure 2b), while etching revealed a new component at 398.0 eV characteristic of Zn–N interaction (Figure 2c) [58, 59]. These depth‐resolved XPS results are fully consistent with the DFT predictions that polyol oxygen and triazole nitrogen motifs serve as preferential Zn^2+^ coordination sites. Importantly, the spatial localization of Zn–O and Zn–N species within the interior of the interphase indicates that Zn^2+^ transport occurs through a coordinated pathway embedded in the FCSI scaffold. This coordinated transport mechanism underpins the fixed‐charge‐selective nature of the interphase.

The mechanistic shift from cation conduction via anion motion to hopping through fixed charge is directly reflected in the current–voltage characteristics (Figure 2d) [60]. In neat electrolytes, the current response is strongly dependent on electrolyte composition, showing a transport regime in which both cations and anions must participate to dynamically restore electroneutrality under bias. The resulting current–voltage curves reveal two transport regimes (Figure 2d). At high overpotentials (|V| > 40 mV), the current grows linearly with slope order Zn(OTf)_2_> ZnCl_2_> ZnSO_4._ Near open circuit (|V| < 40 mV), all electrolytes exhibit a pronounced current plateau because the driving force is insufficient for continuous Zn^2+^ drift until counter‐anions back‐migrate to restore electroneutrality. Such anion‐dependent behavior is characteristic of interfaces governed by mobile‐ion redistribution and is inherently susceptible to extended space‐charge formation under confinement [61, 62]. In contrast, the FCSI‐regulated interface exhibits that the plateau shrinks and the overall current response becomes nearly linear. This behavior indicates that charge transport is dominated by cation redistribution within the interphase [63], while anion participation is strongly suppressed as the electrostatic role of mobile anions is replaced by immobile negative charges embedded in the FCSI scaffold.

The ability of fixed charges in transporting cations is quantitatively established by conductometric titration measurements (Figure S12). The conductivity profile exhibits three distinct regimes (Figure S12). Initially, conductivity decreases as dissociated H^+^ ions from pre‐added HCl are neutralized. In this stage, pH increases slightly [64]. The second regime is a conductivity plateau, indicating the completion of H^+^ neutralization and the onset of NaOH titration of the FCSI backbone upon Na^+^ coordination. Here, pH rises more sharply, consistent with OH^−^ release during cation coordination. In the final regime, both conductivity and pH increase rapidly, dominated by excess OH^−^. The conductivity plateau corresponds to a buffering capacity of ∼3 mAh, signifying a large reservoir of exchangeable cationic sites. This ion‐exchange capacity defines the maximum amount of charge that can be locally compensated without involving long‐range anion migration. The magnitude of this capacity demonstrates that the interphase contains a sufficiently large reservoir of immobile negative charges and coordination‐active sites to sustain a Zn^2+^‐rich environment.

Having established both the transport behavior and the intrinsic charge‐compensation capacity of the interphase, we next consider the spatial consequence of this substitution. Dielectric loss spectra reveal that the FCSI establishes steep Zn^2+^ concentration gradients across the transport pathway (Figure 2e). In the absence of FCSI, electroneutrality must be dynamically reconstructed over an extended ionic diffusion length through mobile ions, leading to broad concentration gradients and the expansion of space‐charge layers into the electrolyte. By contrast, FCSI confines charge compensation to a narrow interfacial region: a more than 10‐fold increase in concentration, along with approximately a hundredfold reduction in the distance between the high‐concentration region and the electrode. Immobile negative charges provide static electrostatic screening, while the Zn^2+^ reservoir ensures local charge balance without requiring anionic redistribution. This enrichment is further supported by a higher electric double‐layer capacitance at the Zn surface (Figure S13), confirming that the interphase concentrates active ions. As a result, the effective ionic diffusion length is reduced, and the spatial extent of space‐charge layers is intrinsically limited.

**FIGURE 3:**
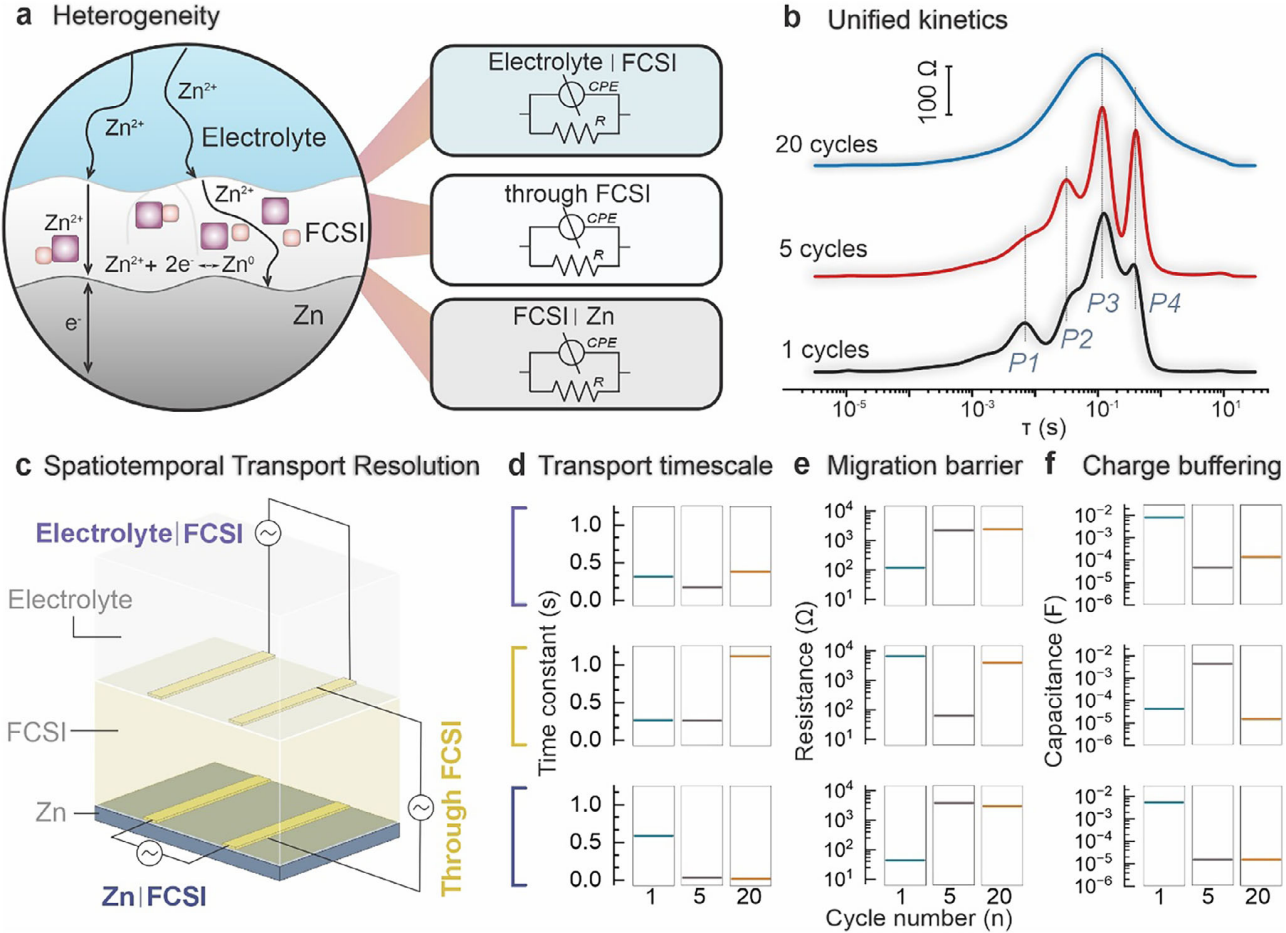
Spatiotemporal transport synchronization. (a) Schematic of Zn deposition/stripping across the electrolyte|FCSI|Zn stack with corresponding equivalent circuit elements (*R*, resistance; CPE, constant phase element). (b) DRT spectra over cycling. (c) Schematic of the configuration used for impedance measurements able to isolate each interface: electrolyte|FCSI, through‐FCSI, and FCSI|Zn. (d–f) Evolution of (d) time constants, (e) resistances, and (f) capacitances for the three regions over cycling.

The spatial confinement of charge compensation established above fundamentally changes not only where charge is balanced but also how interfacial processes evolve in time. In conventional Zn interfaces governed by mobile‐anion‐mediated electroneutrality, multiple electrochemical processes, including ion migration, charge transfer, and interfacial polarization, operate over disparate timescales and compete locally, giving rise to pronounced kinetic heterogeneity. Introducing FCSI does not eliminate interfacial heterogeneity; rather, it renders it structurally explicit by creating two well‐defined phase boundaries: electrolyte|FCSI and FCSI|Zn (Figure 3a). The resistive (*R*) and constant phase element (CPE) components can be used as phenomenological descriptors of distinct interfacial transport processes. R represents the effective barrier associated with ion migration and charge transfer across a given interfacial region, while the CPE captures non‐ideal capacitive behavior arising from distributed charge accumulation and interfacial polarization under confinement [65]. At the electrolyte|FCSI boundary, *R* reflects the resistance to Zn^2+^ entry into the FCSI interphase, whereas the associated CPE represents charge accumulation and dielectric response within the interphase matrix. In FCSI and at the FCSI|Zn interface, R is dominated by Zn^2+^ de‐coordination and electron‐transfer kinetics at the metal surface, while the corresponding CPE captures interfacial charge buffering enabled by coordinated Zn^2+^ species.

The distribution of relaxation times (DRT) provides a direct temporal decomposition of how these spatially distinct processes respond under electrochemical operation [66, 67]. At early cycles (Figure 3b), the DRT spectra resolve multiple well‐separated relaxation processes (P1–P4), spanning over several orders of magnitude in characteristic time. The widespread of these timescales reveals a kinetically fragmented interface in which outer polarization, interphase transport, and charge transfer operate independently. The fastest process (P1) is assigned to outer interphase polarization and space‐charge compensation at the electrolyte|FCSI boundary, consistent with rapid electrostatic rearrangement under locally non‐electroneutral conditions. Intermediate processes (P2–P3) correspond to ion migration within the FCSI and reaction‐coupled charge transfer at the inner (FCSI|Zn) boundary, revealing the sequential coordination, transport, and de‐coordination of Zn^2+^. The slowest process (P4) is attributed to long‐range concentration relaxation or interphase structural reorganization that remains kinetically decoupled from the primary electrochemical reaction.

With continued cycling, the DRT spectra evolve toward a markedly different regime. After five cycles, the interface undergoes a pronounced reorganization. The attenuation of P1 indicates stabilization of the near‐surface electrostatic environment, as the permittivity profile and counter‐ion distribution become more uniform. P2 sharpens and intensifies, suggesting that coordination environments converge toward a single dominant transport barrier once binding sites are homogenized and the FCSI network reaches a steady swelling state. Meanwhile, the clear separation between P3 and P4 reflects emerging kinetic stratification, where bulk transport through the interphase becomes decoupled from slower boundary relaxation processes following stabilization of the FCSI|Zn interface. After extended operation (20 cycles), the initially distinct relaxation modes merge into a single, broadened peak centered at ∼0.1 s. Notably, this convergence involves both the acceleration of initially slow processes (P3, P4) and the modest deceleration of initially fast ones (P1): the fastest processes slow as the outer boundary densifies into a stabilized electrostatic boundary condition, while the slowest processes accelerate as Zn^2+^ percolation and coordination pathways mature in the interphase interior. The result is not uniform acceleration but kinetic synchronization [68]. In other words, the FCSI progressively compresses the transport hierarchy into a unified relaxation process, effectively suppressing the slow relaxation modes associated with long‐range anion redistribution and space‐charge equilibration that would otherwise dominate interfacial dynamics under confinement. The resulting unified timescale does not reflect an acceleration of the intrinsic Zn^2+^ charge‐transfer process, but rather the removal of parasitic bottlenecks that decouple interfacial processes and limit overall reversibility.

While the DRT analysis reveals the temporal hierarchy of interfacial processes, it provides only a global projection of relaxation dynamics and does not uniquely resolve their spatial origin within the multilayer interface. In particular, the coexistence of electrolyte|FCSI and FCSI|Zn boundaries implies that similar time constants may arise from fundamentally different transport environments. To overcome this limitation, we developed an impedance spectroscopy platform incorporating four passivated electrodes—two positioned at the top surface and two at the bottom of the FCSI layer, which is supported on a deposited Zn electrode (Figures 3c and S14). This configuration allows us to do spatiotemporal Havriliak–Negami (H–N) analysis, which enables simultaneous characterization of relaxation time, distribution breadth, and capacitive response across spatially distinct interfacial regions [69]. Unlike conventional DRT, which decomposes impedance solely along the time axis, the H–N framework captures continuous relaxation distributions and enables direct mapping between electrochemical kinetics and specific interfacial regions [70]. Each relaxation branch is described by a resistive amplitude (*R*), a characteristic relaxation time (*τ*), and a capacitance (*C* = *τ*/*R*). *R* corresponds to the ease of ionic passage, *C* represents the buffering capacity, and *τ* indicates the transport timescale (Figures 3d–f).

At the electrolyte|FCSI interface (Figures 3d–f, top panels), the characteristic relaxation time *τ* decreases after five cycles despite an increase in resistance *R*. This seemingly counterintuitive behavior originates from a substantial reduction in capacitance *C*, indicating the formation of a dense diffusion layer driven by strong Zn^2+–^FCSI coordination. XPS evidence of Zn–donor interactions confirms that coordinated Zn^2+^ accumulates at the interphase surface (Figure S15). As a consequence, ionic mobility across the boundary decreases, leading to higher resistance, yet the electrostatic equilibration becomes faster due to reduced capacitive buffering. By 20 cycles, a slight increase in *C*, accompanied by a modest rise in *τ* suggests partial narrowing of ion pathways, suggesting progressive densification and mild clogging at the electrolyte‐facing boundary. These trends indicate that the outer interface evolves into a stabilized, dense layer that imposes a fixed electrostatic boundary condition for transport through the rest of the stack.

Within the FCSI interior (Figures 3d–f, middle panels), the dominant evolution reflects the transition from an initially dry and Zn^2+^‐depleted core into a fully wetted and coordination‐enabled transport medium. Between the first and fifth cycles, *R* decreases by orders of magnitude as Zn^2+^ concentration increases and percolation pathways expand. Simultaneously, *C* rises sharply, consistent with solvent infiltration and an increase in dielectric constant (Figure S16). Therefore, early cycles activate previously inactive domains. The associated reduction in *τ* and shift of the loss peak (Figure S16) toward higher frequency signify faster equilibration dynamics [71]. Upon further cycling to 20 cycles, *R* increases again while *C* decreases and *τ* rebounds, reflecting re‐coordination‐induced densification of the FCSI matrix. These changes indicate that once ion pathways are established, the interphase undergoes re‐densification into a compact yet still conductive configuration. The associated modest rebound in *τ* does not signify kinetic degradation: the DRT at 20 cycles remains a single merged peak rather than reverting to separated modes, confirming that the system has reached a synchronized steady state in which all interfacial processes operate within a narrow timescale window.

At the FCSI|Zn interface (Figures 3d–f, bottom panels), the initial state is characterized by low resistance and high capacitance, suggesting a highly permeable and diffuse contact region. After five cycles, strong Zn^2+–^FCSI coordination forms a defined interphase (Figure S17), reducing capacitance while increasing resistance as ions traverse a more coordinated environment. Despite this, *τ* shortens, indicating that overall Zn^2+^ transport becomes fast once a coordinated pathway is established. By 20 cycles, stabilization of R, C, and *τ* to less than 0.1 s regime indicates the emergence of a Zn^2+^‐rich, ionically conductive boundary that maintains steady transport kinetics. The charge transfer resistance is slightly reduced compared to the uncoated electrode, which may undergo passivation (Figure S18) [4, 72, 73, 74, 75].

Across all regions, the H–N analysis reveals a consistent trajectory: an initial conditioning phase marked by spatially fragmented kinetics evolves toward a synchronized transport regime characterized by stabilized boundaries and coordinated ion pathways. The dielectric response further supports this picture, showing progressive Zn^2+^ uptake and the formation of a Zn^2+^‐rich interfacial layer (Figure S16). Importantly, this evolution shows the intrinsic function of the FCSI: by encoding immobile negative charges and coordination‐active sites, the interphase converts mobile‐anion‐mediated electroneutrality into localized charge compensation. This transformation collapses disparate relaxation timescales into a unified dynamic response.

**FIGURE 4:**
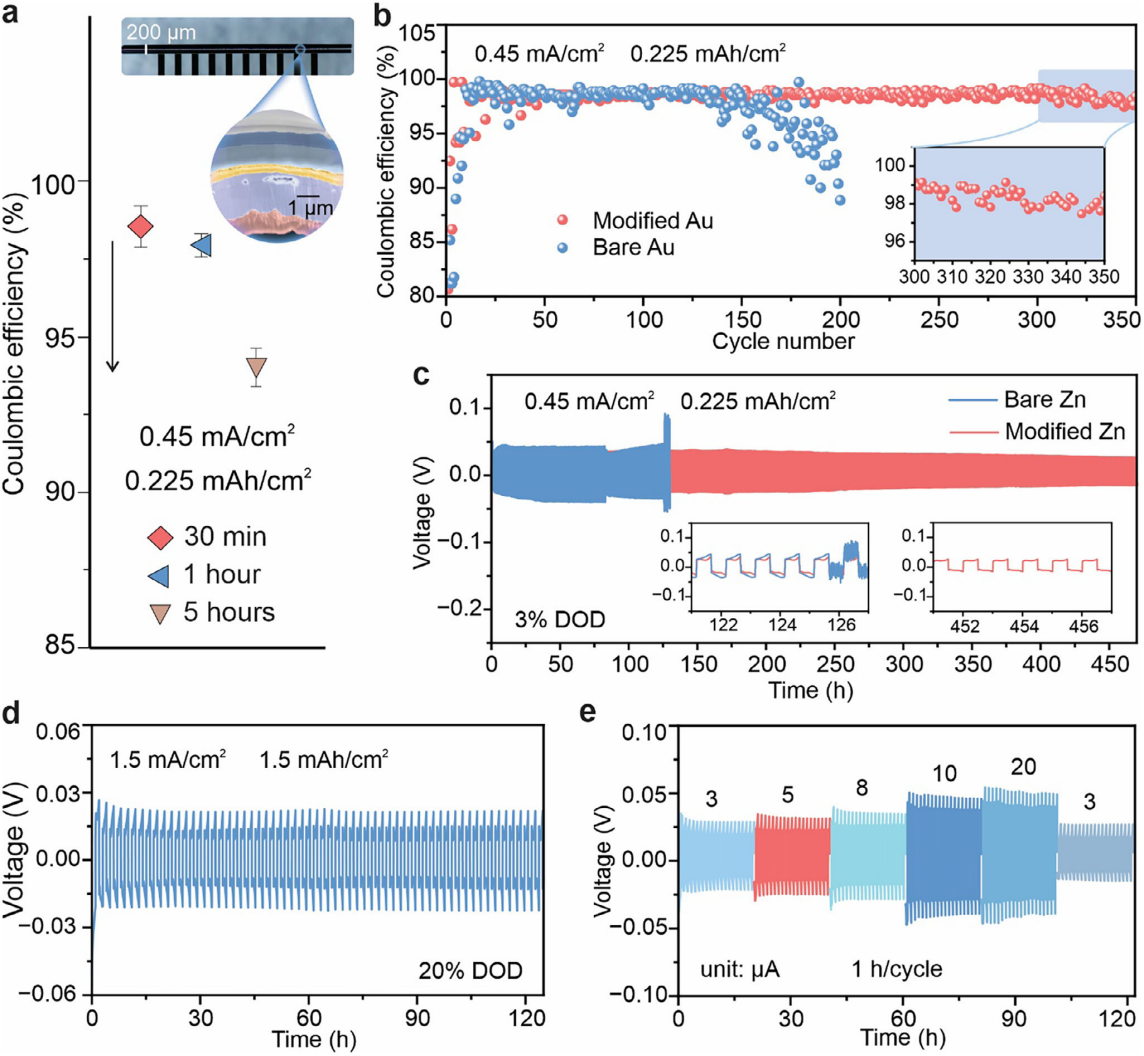
Half‐cell validation. (a) Significantly suppressed rest‐induced Coulombic efficiency loss with FCSI. Inset shows the optical image of the micro‐Swiss‐roll Zn electrode and cross‐sectional SEM image of the layer stack in the microelectrode. (b) Coulombic efficiency of Au microelectrodes with and without FCSI. (c) Voltage profiles of symmetric Zn cells at 1.5 µAh. Insets compare voltage fluctuations over cycling. (d) Long‐term cycling of Zn symmetric cell with FCSI at 10 µAh. (e) Rate performance of Zn symmetric cell with FCSI.

The spatiotemporal transport analysis establishes that the FCSI progressively transforms an initially heterogeneous interface into a dynamically synchronized system governed by localized charge compensation and coordinated Zn^2^^+^ transport. Moroever, by eliminating slow, anion‐mediated relaxation pathways and imposing a fixed electrostatic boundary condition, the interphase suppresses extended space‐charge formation both spatially and temporally. A direct consequence of this transformation is expected to emerge under rest conditions. If charge compensation is truly localized and dynamically synchronized, long‐timescale relaxation pathways should be suppressed, preventing rest‐induced CE collapse. This prediction is directly validated in Figure 4a, where the CE of a micro‐Swiss‐roll electrode comprised of a 1.75 µm Zn layer coated with a 1 µm FCSI (inset in Figure 4a) after extended rest periods (5 h) remains stable, with efficiency losses reduced from ∼40% to ∼5%. Even at a lower electrolyte concentration (1 mol/L), which extends the ESCL, the FCSI maintains a high CE of 97.9% (Figure S19). CE was further tested by depositing Zn onto the Au current collector beneath the FCSI (Figure 4b). At a 1.5 µAh plating capacity, the initial CE without the FCSI is about 80%—well below values for bulk‐flooded cells [76], revealing an intrinsic limitation of deposition under microscale and electrolyte‐limited conditions. While FCSI did not substantially alter the initial CE due to the initial conditioning process as illustrated above, differences emerged after 15 cycles: the CE of the FCSI integrated microelectrode maintained CE > 97.8% for more than 350 cycles, but the unmodified electrode failed after 135 cycles. Galvanostatic cycling profiles confirmed the durability benefits (Figure 4c). At 1.5 µAh capacity, the FCSI enabled stable Zn plating/stripping for over 470 h, compared to only ∼125 h without it, where voltage fluctuations signaled degradation (inset in Figure 4c). Even at a higher capacity of 10 µAh, the FCSI maintained stability for more than 120 h (Figure 4d), whereas the unmodified electrode failed within 54 h (Figure S20). Rate performance remained uncompromised. Symmetric plating/stripping curves were stable at 20 µA, and overpotentials recovered immediately upon returning to lower currents, with no evidence of soft short circuits (Figures 4e and S21).

**FIGURE 5:**
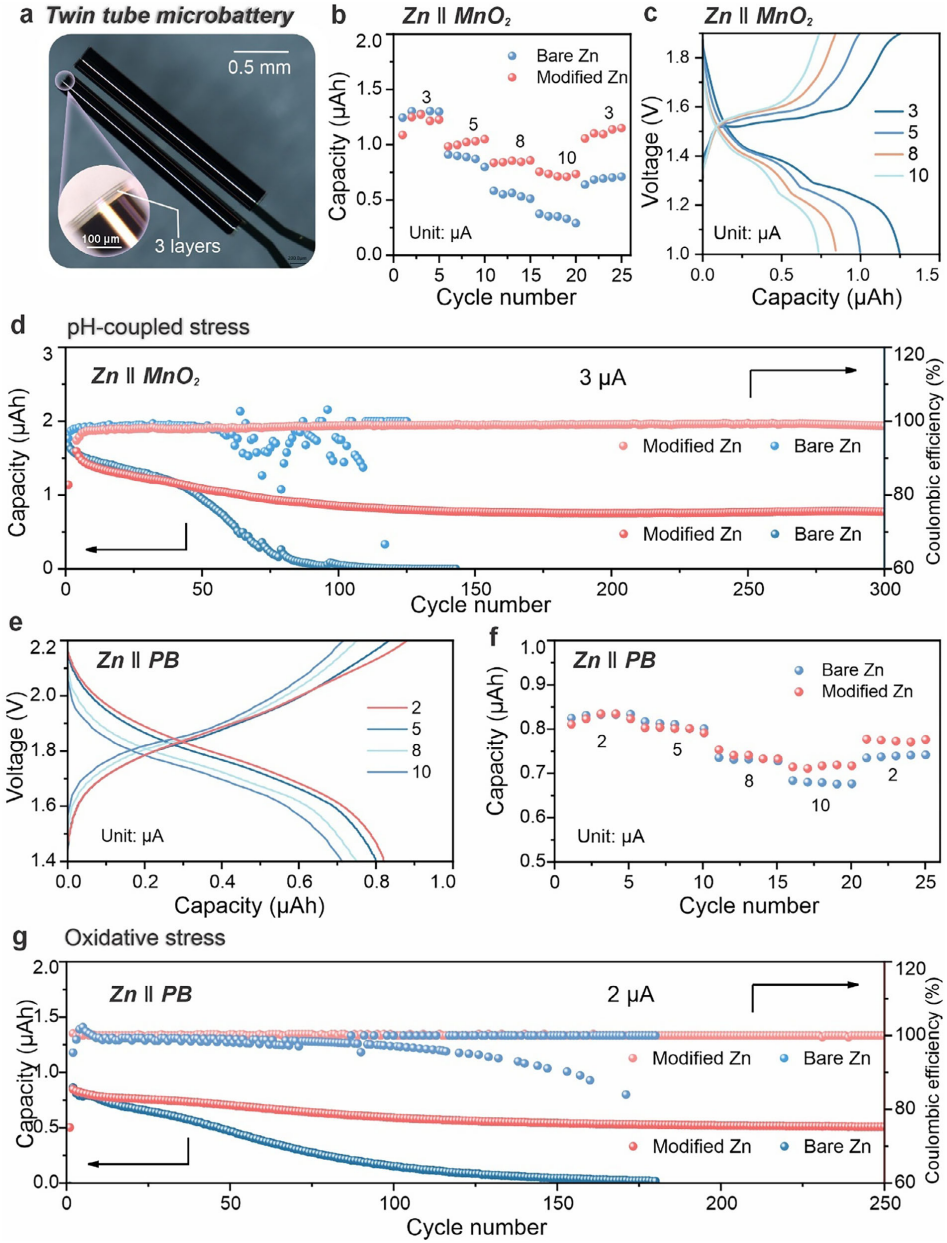
Extreme stress validation. (a) Optical image of a twin micro‐Swiss‐roll microbattery with inset showing the diameter of one micro‐Swiss‐roll electrode (scale bar: 100 µm). (b) Rate performance of Zn||MnO_2_ cells with and without FCSI. (c) Corresponding galvanostatic charge‐discharge profiles of the Zn||MnO_2_ cell with FCSI. (d) Long‐term cycling of Zn||MnO_2_ cell at 3 µA. (e) Galvanostatic charge‐discharge profiles of Zn||PB cell with FCSI at different currents. (f) Rate performance of Zn||PB cells with and without FCSI. (g) Long‐term cycling of Zn||PB cells at 2 µA.

Having established the intrinsic stabilization of Zn microelectrodes enabled by the FCSI, we next evaluate its effectiveness under full‐cell conditions using two cathodes that impose fundamentally distinct electrochemical stresses. Two cathodes were selected to impose fundamentally different electrochemical stresses that are absent in half‐cell measurements and cannot be inferred from them. MnO_2_ was selected as a pH‐coupled system, where proton involvement and interfacial chemistry challenge electrostatic stability, whereas PB represents a high‐voltage insertion cathode that introduces strong oxidative stress [77]. We fabricated a twin‐Swiss‐roll design (Figure 5a) to avoid the direct interference of both electrodes in the same micro‐Swiss‐roll electrode. In this configuration, the cathode and anode are wound into two separate micro‐Swiss‐roll electrodes (each 200 µm in diameter) separated by a 75 µm gap, rather than co‐wound into a single roll. This spatial separation ensures that cathode‐side effects, such as transition‐metal dissolution from MnO_2_ or pH gradients generated during proton‐coupled redox, do not directly contaminate the anode interface within the same confined scroll, allowing unambiguous attribution of performance changes to the anode interphase.

Energy storage in MnO_2_ is widely accepted to proceed via a proton‐assisted Zn^2+^ insertion/conversion mechanism [78, 79]. On the Zn side, this coupled‐ion process induces local acidification during discharge and alkalinization during charge. Both effects accelerate parasitic reactions, including hydrogen evolution and the precipitation of zinc hydroxide sulfate, which progressively degrade the anode interface. Notably, the FCSI remains stable under these pH fluctuations (Figure S22), enabling its application in full‐cell configurations. Cyclic voltammetry of Zn||MnO_2_ full cells with and without the FCSI at 3 mV/s over 0.8 to 2.0 V (Figure S23a) shows distinct cathodic and anodic peaks characteristic of the MnO_2_ redox reactions. In rate performance tests (Figure 5b), both configurations exhibit similar capacities at the initial low current of 3 µA, indicating that at slow rates, the Zn^2+^ supply from the anode is sufficient even without the interphase layer. However, the performance gap widens starting from 5 µA, where the modified Zn anode begins to suffer from increased charge‐transfer resistance and non‐uniform Zn^2+^ flux. At 10 µA, the cell with FCSI delivers 0.73 µAh and recovers to 1.11 µAh upon returning to 3 µA—nearly twice the recovered capacity of the unmodified cell. Across the 3–10 µA range, the FCSI‐modified Zn||MnO_2_ cell also preserves the characteristic two discharge plateaus (Figure 5c), confirming that the energy storage mechanism of MnO_2_ remains intact even under high‐rate operation. The superior high‐rate performance of the FCSI‐included cell can be attributed to the FCSI's ability to form a Zn^2+^‐rich interphase that buffers local pH. This ensures that Zn^2+^ flux remains matched to the rapid redox kinetics at elevated rates for the cathode. The FCSI also markedly extends cycling stability (Figures 5d and S23b), retaining 98.2% capacity after 300 cycles at 3 µA with an average CE of 98.7%. In contrast, the cell without FCSI exhibits rapid CE fluctuations and retains only 45.2% capacity after 60 cycles (Figure S23c). Even at a higher current of 5 µA, the cell with FCSI attains 1.02 µAh after 150 cycles with 98.3% capacity retention (Figure S23d).

Alternatively, PB stores charge through a Zn^2+^ insertion/extraction process within its open cubic framework, with minimal structural distortion and negligible dissolution of active species [80]. This mechanism avoids the pH changes and transition‐metal dissolution associated with MnO_2_, but PB operates at a higher voltage (1.4–2.2 V), which creates a more oxidative environment at the Zn anode. In electrolyte‐limited systems, this elevated potential promotes anion enrichment at the Zn interface and accelerates oxidative surface passivation. CV of Zn||PB micro‐Swiss‐roll cells (Figure S24a) shows redox peaks characteristic of Zn^2+^ insertion/extraction into and from the PB framework, consistent with the literature [81]. Galvanostatic charge–discharge profiles at various current densities display sloped plateaus aligned with the CV peaks (Figure 5e). In rate performance tests (Figure 5f), both configurations perform similarly up to 5 µA. However, at 10 µA, the FCSI‐included cell maintains a higher discharge capacity (0.71 vs. 0.67 µAh). Upon returning to 2 µA, the FCSI‐included cell exhibits a capacity recovery that is better than the cell without the FCSI. Cycling stability tests (Figure 5g) further demonstrate the FCSI's protective role in PB cells. While both configurations deliver similar initial capacities, the cell without FCSI declines from 0.86 µAh to 0.47 µAh after only 50 cycles, accompanied by CE fluctuations (Figure S24b). In contrast, the FCSI‐included cell maintains stable cycling for 250 cycles with a CE approaching 100% (Figure S24c), confirming its ability to suppress passivation and side reactions over extended operation. Here, the FCSI's advantage arises from preventing oxidative passivation and limiting anion crowding at the Zn surface under high‐voltage operation by maintaining a Zn^2+^‐rich environment, thereby preserving Zn^2+^ transport pathways during high‐rate cycling.

## 3 Conclusions

In summary, this work demonstrates that microscale aqueous batteries operate in a space‐charge–dominated regime where the extreme confinement breaks local electroneutrality and destabilizes metal deposition. We address this challenge through a molecularly engineered FCSI that replaces mobile‐anion‐mediated charge compensation with immobile negative charges coupled to a coordinated Zn^2+^ reservoir, enabling localized charge neutrality and synchronized ion transport under extreme confinement in a nanoliter electrolyte (∼45 nL). Operando spatiotemporal transport analysis reveals that initially fragmented interfacial processes merge into a unified kinetic regime, corresponding to the emergence of a solid‐ionic interphase that couples Zn^2+^ migration and charge transfer across the electrolyte|FCSI|metal stack. Implemented in micro‐Swiss‐roll electrodes (0.44 mm^2^ footprint), this electrostatic boundary enables highly reversible Zn^0/2+^ cycling and, importantly, suppresses rest‐induced CE collapse from 40% to 5% after 5 h, directly validating the elimination of long‐timescale space‐charge relaxation. The concept remains effective in full microbatteries (1.05 mm^2^) paired with MnO_2_ and PB cathodes, representing proton‐coupled and high‐voltage electrochemical stresses, sustaining operation beyond 250 h with strong rate capability. These results establish fixed‐charge interphases as a framework for electrostatic boundary engineering in confined electrochemical systems. We note that the coordination‐rich environment within the FCSI may additionally modulate Zn^2+^ desolvation kinetics by distributing ligand‐exchange steps across the interphase thickness, as has been suggested for confined fixed‐charge environments in BPMs and at electrocatalyst interfaces [11]. Disentangling this potential solvation‐kinetic contribution from the dominant electrostatic mechanism established here represents an important direction for future work.

More broadly, the physics underlying this work, that fixed charges modulating ion transport within space‐charge regions connects to a rich body of knowledge across electrocatalysis, BPMs, and nanofluidics that offers opportunities for mutual exchange. Drawing on concepts from various electrochemical applications [1], such as the understanding of how interfacial capacitance influences ion solvation kinetics at BPM junctions, or how polyelectrolyte layers within overlapping double layers regulate selectivity in nanofluidic channels, could guide the rational design of advanced interphases for microscale batteries. Conversely, the spatiotemporal transport analysis presented herein, which elucidates how initially fragmented interfacial processes coordinate during cycling, may provide valuable diagnostic methods for investigating dynamic double‐layer restructuring in electrocatalytic and membrane systems.

## Author Contributions

**Yaping Yan**: methodology, data curation, writing – original draft. **Jiachen Ma**: methodology, data curation, writing – review and editing. **Wenlan Zhang**: methodology, data curation, visualization. **Hongmei Tang**: methodology, data curation, writing – review and editing. **Yue Li**: methodology, data curation, writing – review and editing. **Ruhuai Mei**: methodology, data curation, formal analysis, writing – review and editing. **Yang Huang**: methodology, writing – review and editing, data curation. **Daniil Karnaushenko**: methodology, data curation, writing – review and editing. **Dmitriy D. Karnaushenko**: methodology, data curation, writing – review and editing. **Yumin Luo**: methodology, writing – review and editing. **Kai Zhang**: conceptualization, funding acquisition, supervision, writing – review and editing. **Oliver G. Schmidt**: supervision, writing – review and editing, funding acquisition. **Minshen Zhu**: conceptualization, funding acquisition, methodology, visualization, writing – review and editing, supervision.

## Conflicts of Interest

The authors declare no conflicts of interest.

## Supporting information




**Supporting File 1**: anie73368‐sup‐0001‐SuppMat.docx.

## Data Availability

The data that support the findings of this study are available from the corresponding author upon reasonable request.
