# Supplementary material for: A Fixed‐Charge Interphase Synchronizes Ion Transport to Suppress Space‐Charge‐Driven Inefficiency Under Nanoliter Confinement
Source: Angew Chem Int Ed Engl. 2026 Jun 30;65(36):e5195226. doi: 10.1002/anie.5195226 (PMC13528523; doi:10.1002/anie.5195226)
Supplement: Supplementary file 1 [file ANIE-65-e5195226-s001.docx]

**A Fixed-Charge Interphase Synchronizes Ion Transport to Suppress Space-Charge–Driven Inefficiency under Nanoliter Confinement**

Yaping Yan,^[a,b]^ Jiachen Ma,^[a,b]^ Wenlan Zhang,^[a,b]^ Hongmei Tang,^[a,b]^ Yue Li,^[a,b]^ Ruhuai Mei,^[c]^ Yang Huang,^[d]^ Daniil Karnaushenko,^[a,b]^ Dmitriy D. Karnaushenko,^[a,b]^ Yumin Luo,^[a,b]^ Kai Zhang,^[c]^ Oliver G. Schmidt,^[a,b]^ and Minshen Zhu*^[a,b]^

[a] Y. Yan, J. Ma, W. Zhang, Dr. H. Tang, Yue. Li, D. Karnaushenko, D. D. Karnaushenko, Y. Luo, Prof. Dr. O. G. Schmidt, Dr. M. Zhu

Research Center for Materials, Architectures, and Integration of Nanomembranes (MAIN), Chemnitz University of Technology, Chemnitz 09126, Germany

Email: minshen.zhu@main.tu-chemnitz.de

[b] Y. Yan, J. Ma, W. Zhang, Dr. H. Tang, Yue. Li, D. Karnaushenko, D. D. Karnaushenko, Y. Luo, Prof. Dr. O. G. Schmidt, Dr. M. Zhu

Material Systems for Nanoelectronics, Chemnitz University of Technology, Chemnitz 09107, Germany

[c] Dr. R. Mei, Prof. Dr. K. Zhang

Sustainable Materials and Chemistry, Department of Wood Technology and Wood-based Composites, University of Göttingen, Göttingen 37077, Germany

[d] Dr. Y. Huang

Advanced Materials Thrust, Function Hub, The Hong Kong University of Science and Technology (Guangzhou), Guangzhou 511400, Guangdong, China

Materials and methods

**Swiss-roll microanode fabrication:**

The Swiss-roll microanode was constructed using micro-origami technology, as previously reported.^[1]^ Initially, the glass substrate was sequentially cleaned in acetone, isopropyl alcohol, and deionized water, followed by O_2_ plasma treatment (Pico, Diener electronic GmbH) for 5 minutes at 400 W. After the cleaning of substrate, the sacrificial layer (SL), hydrogel layer (HG), and polyimide layer (PI) were patterned in sequence by repeating a standard photolithography process. The thicknesses of these layers were approximately 300 nm, 800 nm, and 800 nm, respectively. Subsequently, a 10 nm layer of Cr and a 50 nm layer of Au were deposited as the current collector. To precisely control the functional area of the electrode, the connection between the electrode and the contact pad was passivated with an insulator layer of SU-8. The Swiss-roll microelectrode was fabricated by removing SL with an etching solution (3.7 wt.% HCl). In a mixed electrolyte consisting of 2 M ZnSO_4_, 20 g/L H_3_BO_4_, and 0.3 g/L anionic surfactant sodium dodecyl sulfate (SDS), the Zn microanode was electrodeposited using a two-mode chronopotentiometry method, with 100 mA/cm^2^ for the initial nucleation and 20 mA/cm^2^ for the growth of Zn. Finally, utilizing the capillary effect, the Swiss-roll microanode was protected by a fixed-charge selective interphase (FCSI) by placing one drop of polymer solution at the end of the tube.

**Twin Swiss-roll microbattery fabrication:**

Similar to the fabrication process of the Swiss-roll microanode, a three-layer structure and two separate current collectors were initially constructed. Before the MnO_2_ coating, the device was immersed in a solution of 0.01 M SDS, 1 M H_2_SO_4_, and 0.01 M 3,4-ethylenedioxythiophene (EDOT). The PDOT layer was deposited under a constant voltage of 0.9 V using a platinum net and Ag/AgCl (saturated KCl) as the counter and the reference electrode, respectively. Based on the PDOT scaffold, MnO_2_ deposition was carried out in the same three-electrode configuration, using cyclic voltammetry (CV) scans between 0.3 and 0.6 V (vs Ag/AgCl) at a scan rate of 50 mV/s for 12 cycles, followed by a constant voltage of 0.6 V for 90 s. This deposition process was repeated six times in an aqueous solution containing 0.1 M Mn(CH_3_COO)_2_·4H_2_O and 0.1 M Na_2_SO_4_ as the electrolyte. Similarly, the electrodeposition of PB was carried out at a constant voltage in the same three-electrode cell, with the electrolyte containing 0.01 M FeCl_3_, 0.01 M K_3_[Fe(CN)_6_], and 0.05 M KCl. After the deposition of PDOT/MnO_2_ or PB onto the cathode current collector, the same rolling process used for the single microtube was employed to assemble the planar device into a twin tubular device. The Zn layer was then coated onto the current collector scaffold and protected by FCSI.

**Materials characterization:**

XPS measurements were performed with an ESCALAB 250Xi X-ray photoelectron spectrometer (Thermo Scientific) equipped with a monochromatic Al Kα (hν = 1486.7 eV) X-ray source. The morphologies of materials and devices were observed by scanning electron microscopy (DSM982 Gemini and TESCAN) with FIB milling at 30 keV Ga^+^. SAXS experiments on the FCSI dissolved in DMF were carried out at beamline P62 (DESY, Hamburg). The measurements covered a *q*-range of 0.15-6 nm^–1^, with each sample exposed for 30 s and repeated 5 times to exclude beam damage. Scattering contributions from the neat solvent were measured separately and subtracted as background.

**Electrochemical measurement:**

The galvanostatic charge-discharge profiles were recorded using a Neware MHW-200 battery test system. Cyclic voltammetry, electrochemical impedance spectroscopy (EIS), Tafel curve, and chronoamperometry were measured by an electrochemical workstation (MULTIAUTOLAB/M101). The EIS was performed at the open circuit potential with a frequency range of 100 kHz to 0.1 Hz and an amplitude of 5 mV. All those measurements were taken at ambient temperature in open-air conditions. Unless otherwise specified, all electrolytes used in the tests, including ZnSO₄ and others, were at a concentration of 2 M.

**DOD calculation:**

The depth of discharge (DOD) of Zn micro anode is calculated using the following equation:

$$DOD=\frac{Ist}{Mm}$$

Where $I$ (mA/cm^2^) represents the current density applied to the cell during galvanostatic cycling test, $t$ (h) denotes the discharge time, $M$ (mAh/g) corresponds to the theoretical specific capacity of Zn (820 mAh/g).

The mass of Zn ($m$) in the micro anode is determined by:

$$m=V\cdot\rho=h\cdot s\cdot\rho$$

Where $\rho$ refers to the density of Zn (7.14 g/cm^3^), $h$ is the thickness of the Zn microelectrode (1.75 µm). $s$ (0.0484 cm^2^) is the area of the Zn microelectrode, calculated by multiplying the planar length (0.22 cm) and width (0.22 cm) of the micro-Swiss-roll electrode before the rolling-up process. Thus, the active material loading is then calculated as 6×10^-5^ g.

**Complex permittivity calculation:**

A complex impedance is known as

$$Z\left( \omega\right)=Z^{'}(\omega)+j\cdot Z^{''}(\omega)$$

The impedance of a pure capacitive component is written as

$$Z\left( \omega\right)=\frac{1}{j\omega C(\omega)}$$

Pure capacitance is written as

$$C\left( \omega\right)=\varepsilon_{o}\varepsilon_{\gamma}(\omega)\frac{A}{d}$$

Combining the three formulas, the complex permittivity is converted from measured impedance as^[2]^

$$\varepsilon_{\gamma}\left( \omega\right)=\frac{d}{\varepsilon_{o}Aj\omega\left[ Z^{'}\left( \omega\right)+j\cdot Z^{''}\left( \omega\right) \right]}=-\frac{d\left[ Z^{''}\left( \omega\right)+j\cdot Z^{'}\left( \omega\right) \right]}{\varepsilon_{o}A\omega\left| Z\left( \omega\right) \right|^{2}}$$

where $\varepsilon_{o}=8.85\times{10}^{-12}$ F/m is the value of the permittivity of free space, and the geometrical parameters were $A=0.33$ mm^2^ and $d=1$ µm.

**Ion concentration and diffusion length calculation:**

By neglecting the interactions between ions, the ionic conductivity ($\sigma$) can be described as:

$$\sigma=n\cdot e\cdot\mu$$

Where $n$ represents the total ion concentration, $e$ is the elementary charge, and $\mu$ corresponds to ion mobility. Applying the Einstein relation, which links mobility to the diffusion coefficient (D) as:

$$D=\frac{kT}{e}\cdot\frac{1}{\mu}$$

Where $k$ is the Boltzmann constant, and $T$ is the absolute temperature. The ion concentration can then be expressed as:

$$n=\frac{\sigma}{D}\cdot\frac{kT}{e^{2}}$$

Here, $\sigma$ is obtained from the dielectric loss spectra according to $\sigma=\varepsilon_{0}\cdot{\varepsilon_{\gamma}}^{''}(\omega)\cdot\omega$. The diffusion coefficients for the FCSI and electrolyte are estimated as 1.2·10^-11^ m^2^/s and 3.8·10^-10^ m^2^/s, respectively.^[3,4]^ Furthermore, the Diffusion length is expressed by equation: $L=\sqrt{D/\pi\cdot f}$, where $f$ is the modulation frequency.

**DFT calculation:**

All the first-principles calculations were carried out using Gaussian under the DFT framework. The widely used hybrid functional B3LYP was adopted.^[5,6]^ The high-accuracy basis set of 6-311++G (d, p) with GD3BJ dispersion correction was employed to provide a more accurate description of the electronic structure.^[7,8]^ All calculations of molecules and ions were performed in a nonsolvent environment and with a spin multiplicity of 1.

**Figure S1** Cross-sectional scanning electron microscopy (SEM) image of the Zn layers in the micro-Swiss-roll electrode.

**Scheme S1** Synthesis pathways to (a) the glucose-derived monomer **9**, (b) the functionalized PEG **10**, and (c) the interphase polymer.

**Figure S2** ^1^H nuclear magnetic resonance spectrum for polymer.

The detailed synthetic protocol for the polymer was described in our previous paper.^[9,10]^ The structure of the polymer was characterized by ^1^H nuclear magnetic resonance spectroscopy. As shown in Figure S2, the resonance peaks at 3.7 and 3.4 ppm (green and grey regions, respectively) correspond to the chain methylene group and the methyl group adjacent to oxygen,^[11]^ confirming the presence of the PEG block in the polymer. Furthermore, the signals in the purple region between 7.1 and 7.5 ppm are related to aromatic C–H in the repeating units of the triazole ring and the Benzene ring. The broad signals in the yellow region between 3.8 and 5.2 ppm are assigned to the C–H groups in the glucose ring and the methylene groups in the benzyl,^[10]^ verifying the glucose-derived monomer as the other structural unit of the polymer.

**Figure S3** Fourier transform infrared spectroscopy of the polymer.

The structure of the polymer was further characterized by Fourier-transform infrared spectroscopy (Figure S3). The characteristic stretching vibrations of C–H in the PEG block and C≡C bonds appear at approximately 2870 and 2,080 cm^−1^, respectively.^[12]^ In addition, the multiple signals observed in the range of 500-950 cm^−1^ (purple region) are attributed to the benzyl and triazole groups.^[10]^ More importantly, the –C–O bending vibration and N–N=N asymmetrical stretching vibration are responsible for the peaks at 1087.8 and 3130 cm^−1^, respectively,^[13]^ verifying the presence of abundant oxygen and nitrogen donor sites.

**Figure S4** Small-angle X-ray scattering (SAXS) profile of the FCSI dissolved in DMF.

**Figure S5** Nyquist plots collected with the frequency range of 100 kHz to 1 Hz. (a) Bare Au (inset: enlargement of indicated range). (b) Coated Au (inset: enlargement of indicated range).

The ionic conductivity of the electrolyte was evaluated according to the following equation:

$$\sigma=\frac{L}{R_{b}S_{Au}}$$

where $L$ represents the distance between two Au electrodes, $S_{Au}$ represents the contact area (9 mm^2^), and $R_{b}$ is the resistance according to the EIS measurements. For the system without interphase: $R_{b}=$58.4 Ω. Thus, $\sigma_{Bare}=$ 57.1 mS/cm. For the system with interphase: $R_{b}=$ 59.9 Ω. Thus, $\sigma_{Coated}=$ 55.6 mS/cm.

**Figure S6** (a) Nyquist plot of Au electrode with PVA coating (inset: enlargement of indicated range). Linear polarization curves of Zn microelectrodes coated with (b) PVA and (c) FCSI, compared with (d) the bare Zn microelectrode.

**Figure S7** Molecular orbitals of the glucose-derived core and its Zn^2+^ complex calculated by DFT.

**Figure S8** (a) Comparison of the Zn^2+^ transference number. Chronopotentiometry under a polarization potential of 10 mV for 3000 seconds and Nyquist plots before and after chronopotentiometry measurements for (b) bare Zn and (c) coated Zn cells with ZnSO_4_ electrolyte.

**Figure S9** (a) Electronic conductivity and (b) ionic conductivity of FCSI over cycling.

**Figure S10** Cyclic voltammetry of bare FCSI.

**Figure S11** Zn LMM Auger at the polymer|electrolyte interface after stripping 25 mAh/cm^2^ Zn.

**Figure S12** Simultaneous conductometric and potentiometric titration of the FCSI.

**Figure S13** CV tests of symmetrical micro cells with (a) bare and (c) coated Zn electrodes at different sweep rates, corresponding to (b, d) plots of capacitance currents versus scan rate.

According to the previous report,^[14]^ the EDLC value was calculated using the equation: $C=\frac{i}{v}$, where $C$ means the electric double-layer capacitance, $i$ corresponds to the current and was determined by half of the difference between positive and negative scanning currents under each scanning rate.

**Figure S14** Optical microscope image of the device used for impedance spectroscopy measurements.

**Figure S15** XPS spectra of (a) Zn LMM Auger, (b) N 1s, (c) O 1s, and (d) C 1s of the polymer surface after cycling.

The interaction between Zn^2+^ and surface donor sites of the polymer was investigated using XPS. As depicted in Figure S15a, the Zn LMM spectrum reveals the clear coordination of Zn^2+^ with both O (495.5 eV) and N (497.5 eV) in the polymer chains, consistent with the corresponding N 1s and O 1s spectra. The N 1s spectrum exhibits four splitting peaks at 401.3, 400.2, 399.3, and 398.6 eV, corresponding to N═N, N─N, C─N, and their coordination with Zn^2+^ within the triazole group (Figure S15b). The O 1s spectrum further confirms the Zn-O coordination via the subpeak at 530.9 eV (Figure S15c). The coordination also affects the core electrons of atoms near donor sites, resulting in a distinct satellite peak at 288.5 eV in the C 1s spectrum, assigned to C-O-Zn/C-N-Zn (Figure S15d).

**Figure S16** Dielectric loss spectra as a function of frequency for the (a) electrolyte|FCSI, (b) through FCSI, and (c) FCSI|Zn configurations after 1, 5, and 20 cycles.

Dielectric loss spectra were derived from the complex impedance to make a more resolved analysis of ionic transport and relaxation dynamics for the electrolyte|FCSI, through FCSI, and FCSI|Zn configurations over cycling. As shown in Figure S16, with increasing cycling numbers, the dielectric-loss spectra of all three configurations display an overall increasing trend across the entire frequency window, indicating a wetting process along the Zn^2+^-permeable pathway. Eventually, their dielectric relaxations converge to nearly the same high level after 20 cycles. Under this situation, almost all the donor sites in the FCSI segments are activated to participate in coordination polarization with Zn^2+^, rendering the polymer matrix effectively homogeneous for Zn^2+^ conduction.^[15]^ Notably, the FCSI interior experienced a more prolonged evolution over cycling (Figure S16b). This behavior can be attributed to the fact that the uptake of both solvent molecules and Zn^2+^ ions initiates from the configurations shown in Figure S16a and S16c, which leads to a delayed development of polarization in through FCSI relative to the other two systems. Furthermore, this progressive diversification in polarization allows a faster relaxation for Zn^2+^ migration,^[16]^ as evidenced by the shift of the dielectric-loss shoulder toward lower frequencies.

**Figure S17** XPS spectra of FCSI after cycling and etching to ~200 nm: (a) Zn LMM Auger, (b) N 1s, (c) O 1s, and (d) C 1s.

XPS was employed to investigate the interaction between Zn^2+^ and the donor sites of the FCSI. After etching the FCSI layer to ~200 nm, the Zn LMM spectrum (Figure S17a) reveals the distinct coordination of Zn^2+^ with both O (495.5 eV) and triazole N (497.5 eV) in the polymer chain. This observation is consistent with the splitting peaks of Zn-N (398.5 eV) and Zn-O (530.6 eV) in N 1s and O 1s spectra, respectively (Figure S17b and S17c). Furthermore, the strong Zn^2+^–FCSI coordination induces an increase in binding energies of C-O/C-N bonds, giving rise to an additional satellite peak at 288.7 eV in the C 1s spectrum (Figure S17d), corresponding to C-O-Zn/C-N-Zn species.

**Figure S18** Nyquist plots of EIS measurements for Zn microelectrodes in a symmetric cell (a) before and (b) after 20 cycles.

**Figure S19** Comparison of rest-induced Coulombic efficiency loss for microelectrodes with and without polymer interphase in 1M ZnSO_4_.

**Figure S20** Galvanostatic cycling of Zn symmetric cell without FCSI.

**Figure S21** Rate performance of Zn symmetric cell without FCSI.

**Figure S22** pH-dependent stability of the polymer interphase. Nyquist plots of Au microelectrodes coated with (a) pristine FCSI and FCSI treated in (b) NaOH (pH=10), and (c) HCl (pH=3). Insets show the enlarged profiles. (d) CE comparison of the above microelectrodes when each is paired with Zn foil.

**Figure S23** (a) CV curves of Zn||MnO_2_ cell at a scanning rate of 3 mV/s. Galvanostatic charge/discharge curves of the (b) FCSI -coated and (c) bare Zn||MnO_2_ cells at different cycles under a current of 3 µA. (d) Cycling performance at 5 μA.

Figure S23a shows the cyclic voltammetry curves of Zn||MnO_2_ full cells with and without the FCSI. In the cathodic process, two peaks are observed at 1.34 and 1.15 V. Meanwhile, two peaks at approximately 1.65 and 1.78 V in the anodic process correspond to oxidation of manganese. The CV curves are consistent with previous reports,^[17]^ and the results are also in good agreement with the two pairs of plateaus in galvanostatic charge/discharge curves (Figure S23b and S23c). In Figure S23b, the FCSI enhances the capacity retention by 0.78 µAh even after 300 cycles at 3 μA. In contrast, the cell without FCSI exhibits rapid capacity decay, with almost no obvious discharge plateau observed after 100 cycles (Figure S23c). Even at a higher current of 5 μA, the effectiveness of FCSI is demonstrated. As shown in Figure S23d, the cell with FCSI delivers a capacity of 1.02 µAh after 150 cycles, corresponding to 98.3% capacity retention.

**Figure S24** (a) CV curves of Zn||PB cell with and without FCSI. Galvanostatic charge/discharge curves of the Zn||PB cells (b) without and (c) with FCSI at different cycles under a current of 2 µA.

Figure S24a presents the similar CV curves of Zn||PB cells with and without FCSI, indicating the negligible influence of FCSI on the Zn^2+^ insertion/extraction kinetics within the open 3D framework of the PB cathode. During the initial cycles, both cells (Figure S24b and S24c) exhibit nearly identical charge and discharge voltage plateaus at approximately 1.85 and 1.7 V, respectively, further confirming that FCSI has minimal impact on the electrochemical mechanism. However, upon extending the cycling number to 100, the capacity of the bare cell rapidly decreased from 0.86 to 0.15 μAh, eventually approaching 0 μAh, suggesting complete failure of the cell. In contrast, the cell with FCSI maintains a stable capacity, decreasing only marginally from an initial 0.84 μAh to 0.59 μAh after 100 cycles, and retaining 0.52 μAh even after 200 cycles. These results clearly demonstrate the excellent compatibility and stabilizing effect of FCSI under the high operating voltage of the PB cathode.

**References:**

[1]	D. Karnaushenko, D. D. Karnaushenko, D. Makarov, S. Baunack, R. Schäfer, O. G. Schmidt, “Self-assembled on-chip-integrated giant magneto-impedance sensorics” *Adv. Mater.* **2015**, *27*, 6582–6589.

[2]	A. Munar, A. Andrio, R. Iserte, V. Compañ, “Ionic conductivity and diffusion coefficients of lithium salt polymer electrolytes measured with dielectric spectroscopy” *J. Non-Cryst. Solids* **2011**, *357*, 3064–3069.

[3]	Y. Awakura, T. Doi, H. Majima, “Determination of the diffusion coefficients of CuSO_4_, ZnSO_4_, and NiSO_4_ in aqueous solution” *Metall Trans B* **1988**, *19*, 5–12.

[4]	C. Gainaru, E. W. Stacy, V. Bocharova, M. Gobet, A. P. Holt, T. Saito, S. Greenbaum, A. P. Sokolov, “Mechanism of conductivity relaxation in liquid and polymeric electrolytes: direct link between conductivity and diffusivity” *J. Phys. Chem. B* **2016**, *120*, 11074–11083.

[5]	P. J. Stephens, F. J. Devlin, C. F. Chabalowski, M. J. Frisch, “Ab initio calculation of vibrational absorption and circular dichroism spectra using density functional force fields” *J. Phys. Chem.* **1994**, *98*, 11623–11627.

[6],	Gaussian 09, Revision A.02, M. J. Frisch, G. W. Trucks, H. B. Schlegel, G. E. Scuseria, M. A. Robb, J. R. Cheeseman, G. Scalmani, V. Barone, G. A. Petersson, H. Nakatsuji, X. Li, M. Caricato, A. Marenich, J. Bloino, B. G. Janesko, R. Gomperts, B. Mennucci, H. P. Hratchian, J. V. Ortiz, A. F. Izmaylov, J. L. Sonnenberg, D. Williams-Young, F. Ding, F. Lipparini, F. Egidi, J. Goings, B. Peng, A. Petrone, T. Henderson, D. Ranasinghe, V. G. Zakrzewski, J. Gao, N. Rega, G. Zheng, W. Liang, M. Hada, M. Ehara, K. Toyota, R. Fukuda, J. Hasegawa, M. Ishida, T. Nakajima, Y. Honda, O. Kitao, H. Nakai, T. Vreven, K. Throssell, J. A. Montgomery, Jr., J. E. Peralta, F. Ogliaro, M. Bearpark, J. J. Heyd, E. Brothers, K. N. Kudin, V. N. Staroverov, T. Keith, R. Kobayashi, J. Normand, K. Raghavachari, A. Rendell, J. C. Burant, S. S. Iyengar, J. Tomasi, M. Cossi, J. M. Millam, M. Klene, C. Adamo, R. Cammi, J. W. Ochterski, R. L. Martin, K. Morokuma, O. Farkas, J. B. Foresman, and D. J. Fox, Gaussian, Inc., Wallingford CT, **2016**.

[7]	S. Grimme, S. Ehrlich, L. Goerigk, “Effect of the damping function in dispersion corrected density functional theory” *J. Comput. Chem.* **2011**, *32*, 1456–1465.

[8]	S. Grimme, J. Antony, S. Ehrlich, H. Krieg, “A consistent and accurate ab initio parametrization of density functional dispersion correction (DFT-D) for the 94 elements H-Pu” *J. Chem. Phys.* **2010**, *132*, 154104.

[9]	Y. Yan, R. Mei, J. Ma, Y. Huang, Y. Zhu, Z. Lang, C. Li, H. Tang, W. Zhang, J. Lu, O. G. Schmidt, K. Zhang, M. Zhu, “Modular design of functional glucose monomer and block co‐polymer toward stable Zn anodes” *Small* **2024**, *20*, 2400292.

[10]	P. Fuchs, P. Vana, K. Zhang, “Copper(I)-catalyzed azide-alkyne cycloaddition-assisted polymerization of linear glucose-derived co/polymers” *J. Polym. Sci.* **2020**, *58*, 1535–1543.

[11]	J. Viéville, M. Tanty, M.-A. Delsuc, “Polydispersity index of polymers revealed by DOSY NMR” *J. Magn. Reson.* **2011**, *212*, 169–173.

[12]	U. Riaz, S. M. Ashraf in *Characterization of Polymer Blends*, John Wiley & Sons, Ltd, **2014**, pp. 625–678.

[13]	G. Nong, S. Chen, Y. Xu, L. Huang, Q. Zou, S. Li, H. Mo, P. Zhu, W. Cen, S. Wang, “Artificial photosynthesis of oxalate and oxalate-based polymer by a photovoltaic reactor” *Sci Rep* **2014**, *4*, 3572.

[14]	M. Yan, N. Dong, X. Zhao, Y. Sun, H. Pan, “Tailoring the stability and kinetics of Zn anodes through trace organic polymer additives in dilute aqueous electrolyte” *ACS Energy Lett.* **2021**, *6*, 3236–3243.

[15]	S. Das, A. Ghosh, “Charge carrier relaxation in different plasticized PEO/PVDF-HFP blend solid polymer electrolytes” *J. Phys. Chem. B* **2017**, *121*, 5422–5432.

[16]	T. Dam, S. S. Jena, D. K. Pradhan, “The ionic transport mechanism and coupling between the ion conduction and segmental relaxation processes of PEO_20_ -LiCF_3_SO_3_ based ion conducting polymer clay composites” *Phys. Chem. Chem. Phys.* **2016**, *18*, 19955–19965.

[17]	H. Pan, Y. Shao, P. Yan, Y. Cheng, K. S. Han, Z. Nie, C. Wang, J. Yang, X. Li, P. Bhattacharya, K. T. Mueller, J. Liu, “Reversible aqueous zinc/manganese oxide energy storage from conversion reactions” *Nat Energy* **2016**, *1*, 16039.
